# Uncommon Manifestation of Basal Cell Carcinoma on the Fingers: A Case Report and Review of the Existing Literature

**DOI:** 10.1155/crdm/9392963

**Published:** 2026-01-31

**Authors:** Bhakinai Temnithikul, Piyakan Limtanyakul, Teerasit Viyanant, Phyo Zaw Aung

**Affiliations:** ^1^ Department of Dermatology, School of Anti-Aging and Regenerative Medicine, Mae Fah Luang University, Bangkok, Thailand, mfu.ac.th; ^2^ HRH Princess Maha Chakri Sirindhorn Medical Center, Mueang Nakhon Nayok District, Nakhon Nayok, Thailand; ^3^ Department of Dermatology, HRH Princess Chulabhorn College of Medical Science, Bangkok, Thailand; ^4^ Department of Anatomical Pathology, Rajavithi Hospital, Bangkok, Thailand, rajavithi.go.th

**Keywords:** basal cell carcinoma (BCC), digital neoplasm, finger neoplasm, nonmelanoma skin cancer (NMSC)

## Abstract

Basal cell carcinoma (BCC) is a commonly occurring cutaneous malignancy predominantly affecting sun‐exposed areas. While the face and neck are typical sites, distal extremity involvement is rare. This case report presents an atypical case of nodular‐type BCC arising on the right ring finger of a 49‐year‐old male with a history of HIV infection and anabolic steroid use. The absence of any significant trauma and familial predisposition to skin cancer in this patient underscores the importance of considering alternative risk factors. A comprehensive literature review revealed that while sun exposure, scarring, and immunosuppression are established risk factors for finger BCC, our case highlights the potential role of occupational and iatrogenic factors, such as hot oil spillage and HIV, in the pathogenesis of this uncommon malignancy. Notably, the thumb is the most frequent site of finger BCC, with the nodular subtype being the predominant histological variant. Surgical excision, often employing Mohs micrographic surgery, remains the gold standard treatment. In this case, wide excision and dorsal metacarpal artery flap reconstruction were performed. This report expands our understanding of the diverse clinical presentation of BCC and emphasizes the need for a thorough evaluation of potential risk factors in atypical cases.

## 1. Introduction

Basal cell carcinoma (BCC) is the most frequently diagnosed type of skin cancer in humans, making up about 75% of all cases [1]. Approximately 85% of BCC cases occur on the face and neck [1]. Interestingly, the occurrence of BCC on the fingers is considered uncommon, with reported incidences ranging from 0.5% to 2.5% [2]. This makes each documented instance an important contribution to understanding this unusual clinical manifestation. This rarity poses diagnostic challenges, as finger lesions can mimic other conditions such as acral lentiginous melanoma, chronic infections, or inflammatory disorders, underscoring the need for heightened clinical suspicion and histopathological confirmation.

The present case is especially interesting due to the patient’s unique constellation of risk factors, including a history of HIV infection, long‐term anabolic steroid use, and an antecedent traumatic event with hot oil spillage on the affected finger. Such factors are not commonly reported in finger BCC cases and may contribute novel insights into the multifactorial pathogenesis of BCC in atypical locations. Furthermore, the successful wide excision and flap reconstruction preserving digit function illustrate practical management considerations in resource‐limited settings where Mohs micrographic surgery (MMS) is unavailable. This case thus expands the clinical spectrum of finger BCC and highlights the importance of comprehensive evaluation of potential risk factors beyond ultraviolet exposure. Additionally, we provide a summary and review of the existing literature regarding BCC on fingers (Table 1).

**TABLE 1:** Details of all published cases of histologically confirmed BCC of the finger.

Case	Age	Gender	Location	Duration of symptoms	Histology subtypes	Treatment	Authors and published year
1	50	M	Middle finger	NM	NM	Removal of the nail	Eisenklam 1931 [3]
2	66	M	Thumb	NM	NM	Radium	Rechou et al. 1932 [4]
3	78	F	Index finger	7 years	Basosquamous	Amputation	Ashby 1956 [5]
4	69	M	Thumb (subungual)	4‐5 years	NM	NM	Nelson 1970 [6]
5	51	M	Ring finger	19 years	NM	Amputation	Alpert et al. 1972 [7]
6	65	F	Thumb (subungual)	4 years	Superficial spreading	Amputation	Hoffman 1973 [8]
7	87	M	Ring finger	3 years	Adenoid	Excision and FTSG	Enna 1978 [9]
8	66	M	Thumb (periungual)	Many years	NM	Mohs micrographic surgery	Robins et al. 1981 [10]
9	36	M	Little finger (subungual)	15 years	Cystic	Excision	Mikhail 1985 [11]
10	59	F	Thumb (subungual)	15 years	Pigmented	NM	Rudolph 1987 [12]
11	84	M	Ring finger (lateral)	5 years	Nodular	Excision and FTSG	Higuchi et al. 1988 [13]
12	70	M	Index finger (dorsal)	10 years	Sclerosing	Not mentioned	West and Berman 1990 [14]
13	74	M	Thumb (dorsal)	3 years	Infiltrative	Mohs surgery and FTSG	Guana et al. 1994 [15]
14	64	M	Index finger (volar)	5 years	Nodular	Amputation	Bhagchandani et al. 1995 [16]
15	85	F	Index finger (dorsal)	7 years	Nodular	Mohs micrographic surgery and SIH	Oriba et al. 1997 [2]
16	62	M	Thumb (periungual)	NM	NM	Mohs micrographic surgery and SIH	Grine et al. 1997 [17]
17	67	F	Little finger	3 years	Pigmented	Radical excision	Kim et al. 2000 [18]
18	81	M	Thumb (dorsomedial)	20 years	Bowenoid	Amputation	Galeano et al. 2002 [1]
19	83	M	Thumb	Many years	Nodular	Mohs micrographic surgery	Martinelli et al. 2006 [19]
20	83	M	Index finger	NM	Superficial	Mohs micrographic surgery	Brasie et al. 2006 [20]
21	70	M	Thumb (dorsal, periungual)	8 months	Infiltrative	Mohs micrographic surgery + FTSG	Forman et al. 2007 [21]
22	58	M	Thumb (periungual)	3 years	NM	Mohs micrographic surgery	Engel et al. 2008 [22]
23	63	F	Ring finger	2 years	Infiltrative	NM	KIM et al. 2009 [23]
24	50	F	Thumb (periungual)	> 1 year	Recurrent BCC	Amputation	Tehrani and Iqbal 2009 [24]
25	64	M	Thumb (periungual)	3 years	NM	Excision + SIH	Sarfati et al. 2010 [25]
26	80	M	Index finger (periungual)	> 1 year	NM	Patient refused treatment	Ko et al. 2011 [26]
27	45	M	Ring finger (dorsoradial)	1 year	Nodular	Excision + FTSG	Yousif et al. 2013 [27]
28	49	M	Ring finger (dorsal)	2 years	Nodular	Wide excision with dorsal metacarpal artery flap	Our case

Abbreviations: FTSG, full‐thickness skin graft; NM, not mentioned; SIH, secondary intention healing.

## 2. Case Presentation

A 49‐year‐old man with Fitzpatrick skin Type IV presented with a two‐year history of a enlarging ulcerative lesion over the dorsum of the right fourth finger between the metacarpophalangeal joint and proximal interphalangeal joint. The lesion is neither painful nor itchy. The patient reported no significant occupational sun exposure, as he was primarily engaged in indoor work as a personal trainer. There was no history of arsenic exposure, including the consumption of well water. Additionally, there was no personal or family history suggestive of basal cell nevus syndrome. However, he reported spilling of a hot oil drop at this site while cooking, and then, this ulcerative lesion appeared. Medical history included a history of HIV infection for 10 years, treated with dolutegravir 50 mg, lamivudine 300 mg, and tenofovir disoproxil fumarate 300 mg daily. He was also taking an anabolic steroid tablet (methandienone) for 6 years for body‐building purposes. He denied any history of injections of steroids, growth hormones, or testosterone.

**FIGURE 1:**
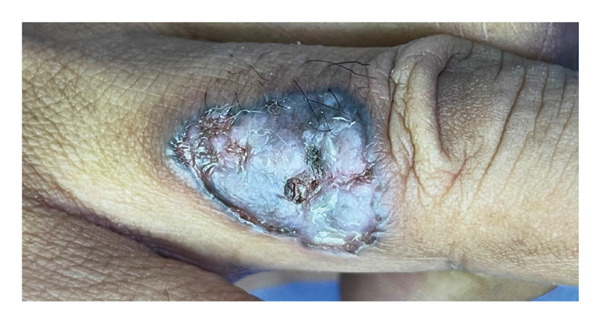
Ulcerative lesion over the dorsum of the right fourth finger between the metacarpophalangeal joint and proximal interphalangeal joint.

**FIGURE 2:**
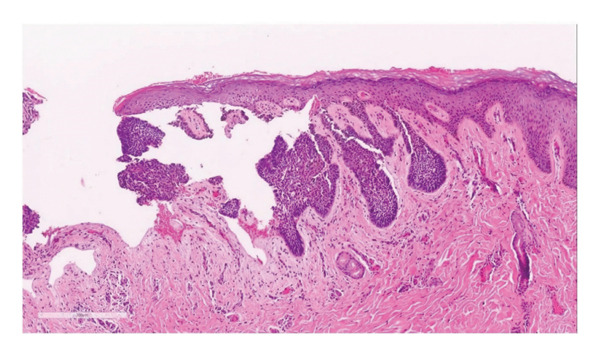
Histopathologic findings: multiple islands of basophilic staining cells with peripheral palisading nuclei and fibromyxoid stroma, extending from the epidermis into the deep dermis.

**FIGURE 3:**
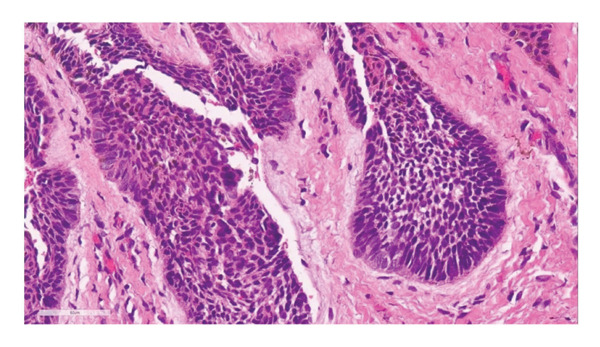
Histopathologic findings (high‐power): proliferation of atypical basaloid cells with peripheral palisading nuclei and fibromyxoid stroma in the upper dermis.

On examination, there was a 3.5 × 2.5 cm ulcerative lesion with a raised, rolled edge. Axillary and epitrochlear nodes were not palpable (Figure 1). The differential diagnoses were pigmented BCC, acral lentiginous melanoma, pyoderma gangrenosum (ulcerative type), and chronic infection, e.g., tuberculosis and nontuberculous mycobacterial infections. Dermoscopy revealed irregular arborizing and serpentine telangiectatic vessels, multiple blue‐gray structureless areas, focal ulceration, and shiny white structures. No pigment network, parallel ridge pattern, or fibrillar pattern was observed. These dermoscopic features were suggestive of BCC rather than acral lentiginous melanoma or inflammatory dermatoses. Biopsy confirmed a nodular type of BCC, showing a proliferation of atypical basaloid cells with peripheral palisading nuclei and fibromyxoid stroma in the upper dermis (Figures 2 and 3). No granulomatous inflammation, necrosis, or organisms were identified. These findings excluded chronic infections, including cutaneous tuberculosis and nontuberculous mycobacterial infections; therefore, additional microbiological cultures and special stains were not performed. The radiographic examination (X‐ray) of the hand revealed no abnormal findings.

**FIGURE 4:**
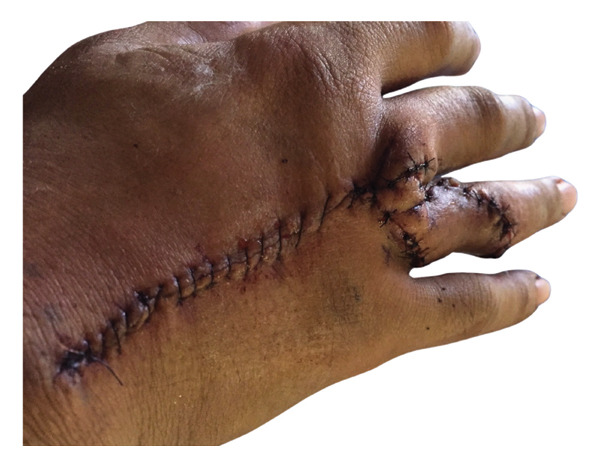
Patient’s hand after the wide excision with dorsal metacarpal artery flap.

**FIGURE 5:**
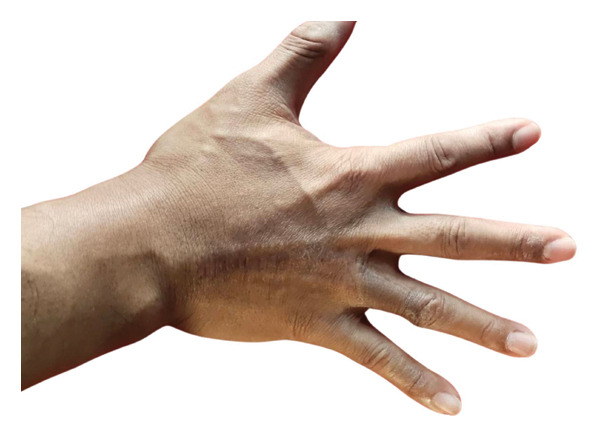
Patient’s hand 18 months after wide excision and reconstruction.

The patient was referred to the plastic surgery department. Then, he was treated with a wide excision (5 mm deep to fascia) followed by reconstruction with a dorsal metacarpal artery flap​ (Figure 4). The postoperative course was uneventful. There was good functional preservation of the digit. One month after surgery, the patient showed no complications and maintained normal digit function. At the most recent 18‐month follow‐up, the wound had healed well with no contracture, no limitation in the range of motion, and no neurological deficits (Figure 5).

## 3. Discussion

BCC involving the fingers is an uncommon clinical entity and poses unique diagnostic and therapeutic challenges. BCC can occur in individuals of various age groups. Based on our comprehensive literature review, as depicted in Table 1, the mean age of onset for BCC is reported to be 67.03 ± 13.01 years. However, our patient, in this context, presents at a comparatively younger age, being 49 years old. Reviewing 28 cases, inclusive of our own, 7 patients were female, while 21 patients were male, 25% and 75%, respectively. Therefore, the prevalence of BCC on fingers appears to be notably higher in the male population.

Regarding risk factors, the pivotal role of sun exposure in BCC development is underscored, particularly in sun‐exposed regions such as the head and neck [15, 23]. The incidence of BCC is higher in geographic regions with intense sunlight and in fair‐skinned individuals who lack melanin protection [1]. Additional contributory factors encompass burns, scars, chronic ulcers, arsenic ingestion, and past radiation exposure [2, 15]. Occupational risk factors, such as radiation exposure during unprotected procedures, have been reported in some cases [1]. Additionally, immunosuppression, xeroderma pigmentosum, and underlying genetically inherited cutaneous disorders can increase the risk of developing BCC [1, 28]. In our specific case, the patient has a history of the accidental spillage of hot oil while cooking, preceding the manifestation of the ulcerative lesion associated with BCC. This would be a potential contributory factor to the development of BCC in his finger. However, the patient denied any history of repetitive trauma to his fingers. Another potential risk factor to consider is the patient’s history of HIV, which has been managed with antiretroviral therapy (ART). Various studies suggest an increased prevalence of nonmelanoma skin cancer (NMSC) in individuals diagnosed with HIV [29–32]. Moreover, the patient’s long‐term use of anabolic steroids is also another potential risk factor. While there is evidence suggesting that systemic corticosteroids may play a role in the development of NMSC [33], the impact of anabolic steroids on NMSC including BCC remains unclear. Therefore, it can be noted that various factors could increase the risk of developing BCC on fingers, and their importance and impact can differ from person to person.

In examining the anatomical distribution of BCC on the fingers, our review (Table 1) highlights a notable predominance of BCC occurrence on the thumb, representing 44.4% of cases. This is followed by the index and ring fingers, each constituting 22.2% of cases. The little finger and middle finger are less commonly affected, with incidences of 7.4% and 3.7%, respectively. Pertinently, our case presented with BCC on the dorsal aspect of the ring finger, aligning with the second most common site of occurrence as established in this literature review. In the context of BCC subtypes, some studies have omitted specifying the exact subtype. Nevertheless, an analysis of 27 case reports in our review reveals a predominance of the nodular subtype (18.5%) in BCC cases on fingers, followed by the infiltrative subtype (11.1%) and the pigmented subtype (7.4%). Additionally, other less common subtypes such as adenoid, basosquamous, bowenoid, cystic, sclerosing, superficial, and superficial spreading were also identified. Notably, the nodular subtype, as the most frequently occurring type of BCC on fingers, aligns with the general prevalence patterns in other body areas [34]. In alignment with these findings, the case under consideration in this study is also classified as a nodular type of BCC.

The management of BCC on digits encompasses a range of treatment strategies, including surgical excision, MMS, full‐thickness skin graft (FTSG), nail plate avulsion, radiation, and curettage combined with topical treatments like podophyllin and salicylic acid. Among these, MMS is particularly effective, especially for BCCs located in the nail unit where conserving tissue and maintaining digit functionality are paramount [19]. FTSG serves as a valuable technique for reconstructing defects following tumor excision. Nail plate avulsion, although less common, is utilized in specific scenarios [19]. Additionally, radiation and topical treatments such as podophyllin and salicylic acid offer alternative therapeutic avenues [21]. As MMS, requiring frozen section analysis, was unavailable in our setting, a wide excision followed by reconstruction with a dorsal metacarpal artery flap was performed for this case. Therefore, this case highlights the versatility of surgical management strategies for BCC in resource‐constrained settings.

In conclusion, this case report, together with a literature review (Table 1), provides a thorough exploration of BCC occurrences on fingers from multiple perspectives. To date, instances of BCC on fingers are relatively rare, primarily documented through case reports and review articles. The etiology and risk factors remain somewhat elusive, lacking definitive clarity. Treatment modalities for BCC on fingers are diverse and should be tailored to the individual patient’s needs. Consequently, there is a distinct need for more comprehensive studies to enhance our understanding and management of this condition in the future.

## Author Contributions

Bhakinai Temnithikul: conceptualization, methodology investigation, writing–original draft, writing–review and editing, and visualization.

Piyakan Limtanyakul: conceptualization, methodology, investigation, project administration, writing–original draft, and writing–review and editing.

Phyo Zaw Aung: conceptualization, methodology, investigation, supervision, writing–original draft, and writing–review and editing.

Teerasit Viyanant: investigation, visualization, and supervision.

## Funding

This study was not supported by any sponsor or funder.

## Ethics Statement

This study adheres to all Ethical Guidelines for human research as outlined in the World Medical Association Declaration of Helsinki. Furthermore, it is exempt from approval by the Mae Fah Luang Ethics Committee on Human Research, indicated by reference number COE 33/2024. As this is a case report involving no more than three cases, the information presented is based solely on a review of medical records and cannot be attributed to any individual without explicit written consent from the patient.

## Consent

Written informed consent was obtained from the patient for publication of this case report and all accompanying clinical and histopathological images. The patient was informed that personal identifiers would not be disclosed.

## Conflicts of Interest

The authors declare no conflicts of interest.

## Data Availability

Data sharing is not applicable to this article as no datasets were generated or analyzed during the current study.
